# A general framework for predicting delayed responses of ecological communities to habitat loss

**DOI:** 10.1038/s41598-017-01070-2

**Published:** 2017-04-20

**Authors:** Youhua Chen, Tsung-Jen Shen

**Affiliations:** 1 Department of Renewable Resources, University of Alberta, Edmonton, T6G 2H1 Canada; 2 Institute of Statistics & Department of Applied Mathematics, National Chung Hsing University, 250 Kuo Kuang Road, Taichung, 40227 Taiwan, ROC; 3 Lab of EcoHealth, School of Health Sciences, Wuhan University, Wuhan, 430072 P.R. China

## Abstract

Although biodiversity crisis at different spatial scales has been well recognised, the phenomena of extinction debt and immigration credit at a crossing-scale context are, at best, unclear. Based on two community patterns, regional species abundance distribution (SAD) and spatial abundance distribution (SAAD), Kitzes and Harte (2015) presented a macroecological framework for predicting post-disturbance delayed extinction patterns in the entire ecological community. In this study, we further expand this basic framework to predict diverse time-lagged effects of habitat destruction on local communities. Specifically, our generalisation of KH’s model could address the questions that could not be answered previously: (1) How many species are subjected to delayed extinction in a local community when habitat is destructed in other areas? (2) How do rare or endemic species contribute to extinction debt or immigration credit of the local community? (3) How will species differ between two local areas? From the demonstrations using two SAD models (single-parameter lognormal and logseries), the predicted patterns of the debt, credit, and change in the fraction of unique species can vary, but with consistencies and depending on several factors. The general framework deepens the understanding of the theoretical effects of habitat loss on community dynamic patterns in local samples.

## Introduction

Habitat destruction, one of the principal factors driving global biodiversity crisis, causes time-lagged, if not instantaneous, loss of species. Such a delayed consequence, described as extinction debt^1–5^, has been increasingly documented in empirical and field studies^6–8^. In addition to debt, ecological processes such as immigration and speciation, also part of the responses that require time to fulfil, can positively contribute to species richness of ecological communities, which are usually termed as immigration credit^3, 4, 9^. However, other than simply counting the delayed loss or gain of species, how will the community structure of species assemblages be altered due to habitat loss^3^? Under what conditions will extinction debt or immigration credit occur in local samples? Therefore, it is necessary to compare the community patterns at equilibriums before and after habitat destruction in order to adequately address these important, but yet unsolved, issues.

Recently, Kitzes and Harte (2015) (hereinafter referred as KH for brevity) developed a novel method to estimate the magnitude of extinction debt or immigration credit from two ecological community patterns, namely regional species abundance distribution (SAD) and species-specific spatial abundance distribution (SAAD). Their method was based on several important assumptions: firstly, local communities in the region are always open to speciation or immigration; secondly, the number of species in a local community is determined jointly by SAD and SAAD; thirdly, regional SAD before habitat loss and after a long run since habitat loss is assumed to be in steady equilibrium; and lastly but most importantly, the whole community or region after reaching new steady state will follow the same parametric SAD curve as the original regional SAD before habitat loss. This assumption states that the underlying regional SAD model will be kept invariant (some specific parameters may be changed).

There are indeed other ways to estimate extinction debts^10–12^. However, the elegancy of KH’s method rests with the fact that it is parsimonious and requires only available information as inputs, including the total number of individuals of all species, the area size of the whole region, and the percent of habitat loss. Moreover, by using this simple information, the fitting of unknown parameters in SAD is very straightforward. In contrast, in many previous methods for modelling extinction debts^11, 12^, some parameters are very difficult to estimate and the corresponding empirical values are rarely available for use. Using Diamond’s biogeographic kinetic model^10^ as an example, the relaxation parameter is key to predict time-dependent extinction of species; however, thus far, few empirical values have been reported for different taxa. Of course, no models are perfect; KH’s model can only be applied to predict extinction debt in the whole remaining intact habitat when the original model is employed. Moreover, the assumptions made by HK’s model are too restricted. Regional SAD may shift after habitat destruction, in particular when the destruction is very intense (for simplicity, this relaxation has not been investigated here. We will show that most post-disturbance patterns could be predicted either from fixed SAD or from shifted SAAD scenarios).

It is clear that KH’s original method only measures the change of regional richness (or gamma diversity). This information is insufficient to provide a complete understanding of the consequences of ecological communities in response to habitat loss. In the local field, it is often observed that local diversity can either increase, decrease, or remain unchanged after habitat destruction and can depend on sampling locations as well^13^, which may either remain consistent or contradictory to the temporal pattern of regional diversity change^14, 15^. How can one resolve this local-regional diversity change paradox? Moreover, after habitat loss, either biotic homogeneity (fraction of unique species declines) or heterogeneity (fraction of unique species increases) may emerge^16–18^. In fact, a growing number of previous studies^13, 15, 19^ have empirically documented these diverse, but distinct, ecological change patterns. However, a parsimonious, tractable, and synthetic framework that can link and explain these temporal ecological phenomena is unavailable until date.

Other than regional species richness, KH’s original method is unable to quantify any other diversity patterns directly, in particular for community structure (local diversity change and the changes in the fraction of unique species). However, by using simple modifications and relaxing some assumptions (SAAD can vary before and after habitat loss), we can demonstrate that KH’s model can be generalised to predict very diverse temporal community change patterns across different spatial scales. By doing so, the generalised statistical framework can perfectly resolve the local-regional diversity change paradox and explain the reason behind the increase or decrease in the local species richness or the fraction of unique species between two local communities after habitat loss.

In the present study, we address the following questions: (1) How many species are subjected to delayed extinction in an intact local community during habitat destruction in other areas? (2) How many rare or endemic species will go extinct or immigrate into the focused local community and how do they contribute to the overall magnitude of extinction debt or immigration credit of the whole local community? (3) How will community structure change after habitat destruction? By answering these questions theoretically using the proposed framework, we can conduct diverse predictions and obtain a relatively complete picture on the delayed responses of ecological communities caused by habitat loss. The general framework takes a big step forward to forecast the temporal biodiversity change and project community structure alteration. The framework can not only assess the effects of habitat destruction but is also identically applicable to evaluate the effects of climate change.

## Materials and Methods

### Predicting delayed species loss and gain for a single local community

**Figure 1:**
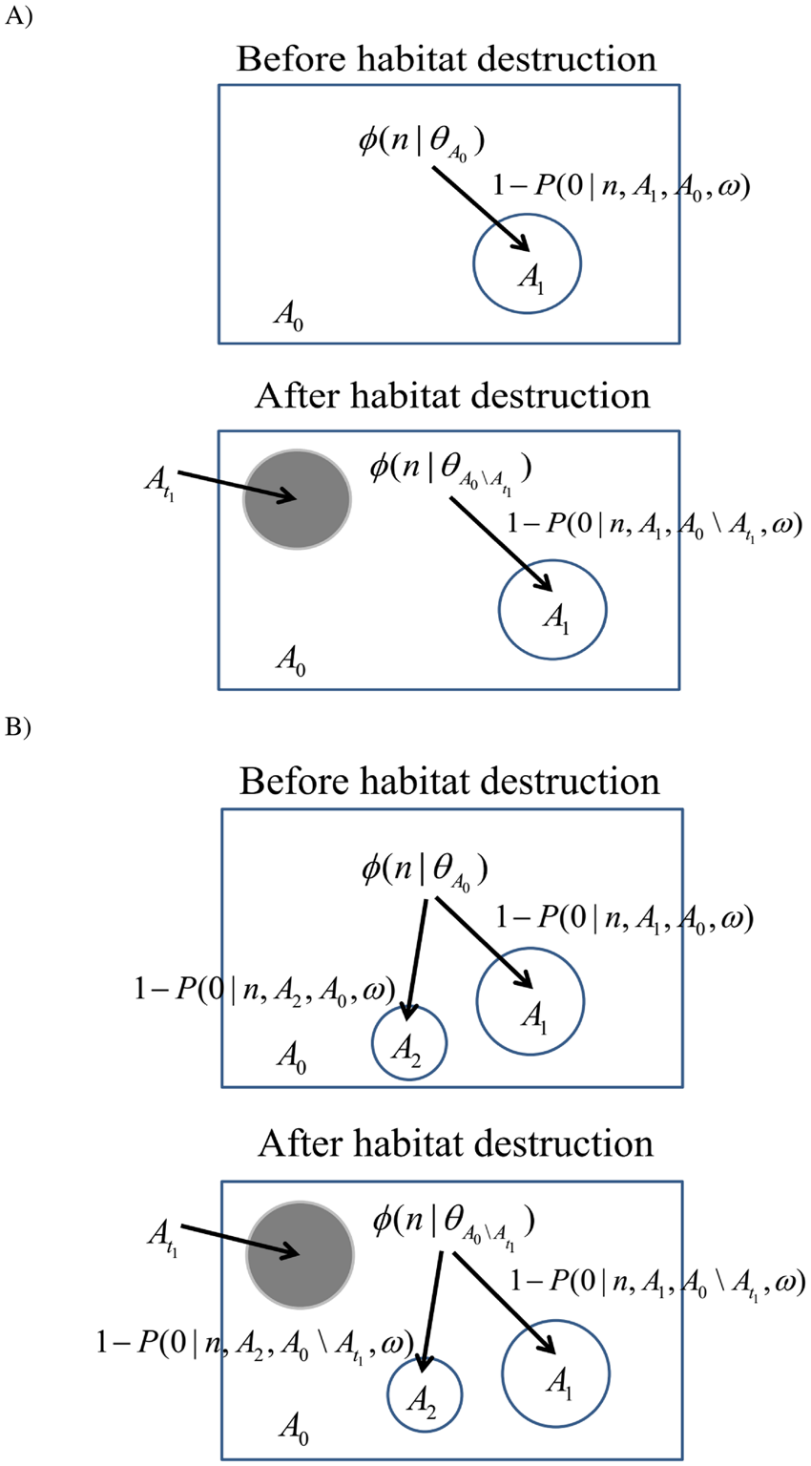
Model configuration for predicting extinction debt in a single local community (**A**) or changes in the fraction of unique species for two non-overlapped local communities (**B**) based on two community patterns, namely regional species abundance distribution and species spatial distribution. Grey area ${A}_{{t}_{1}}$ denotes the destructed area of the region. Species loss and diversity in the circled areas *A*_1_ and *A*_2_ are the intact local communities in the remaining region without habitat destruction. The whole region is not close but open to immigration and speciation.

Suppose there is a region with area size *A*_0_, species number *S*_0_, and total individual number of all the species *N*_0_. Further, there is a local community with area size *A*_1_ that is nested within the region (${A}_{1}\subset {A}_{0}$) (Fig. 1). Then, the number of species found in the local community before habitat loss is given by the product of SAD and SAAD as follows^9, 20, 21^:

1 $${S}_{{A}_{0}}({A}_{1})=\sum _{n}(1-P(0|n,{A}_{1},{A}_{0},\omega ))\varphi (n|{\theta }_{{A}_{0}}),$$

where $\varphi (n|{\theta }_{{A}_{0}})$ represents regional SAD with parameter ${\theta }_{{A}_{0}}$ estimated for the whole region *A*_0_, and $P(0|n,{A}_{1},{A}_{0},\omega )$ represents SAAD with parameter *ω*. Accordingly, the number of endemic species that are only found in the community is given by

2 $${E}_{{A}_{0}}({A}_{1})=\sum _{n}P(n|n,{A}_{1},{A}_{0},\omega )\varphi (n|{\theta }_{{A}_{0}}).$$

In general, in $P(m|n,{A}_{1},{A}_{0},\omega )$, SAAD denotes the probability of observing *m* individuals for a species in the local community *A*_1_, which has a total of *n* individuals in the whole region *A*_0_, which will be explored in detail later. In particular, $\varphi (n|{\theta }_{{A}_{0}})$ here is not a standard probability density function, because $\sum _{n}\varphi (n,{\theta }_{{A}_{0}})={S}_{0}$ ^9^. However, if one utilises a standard probability density function ${\varphi }^{1}(n|{\theta }_{{A}_{0}})$ (thus $\sum _{n}{\varphi }^{1}(n|{\theta }_{{A}_{0}})=1$), then Eq. (1) should be modified accordingly as ${S}_{{A}_{0}}({A}_{1})={S}_{0}\sum _{n}(1-P(0|n,{A}_{1},{A}_{0},\omega )){\varphi }^{1}(n|{\theta }_{{A}_{0}})$.

Suppose there is also another local community with area size ${A}_{{t}_{1}}$ in the region (${A}_{{t}_{1}}\subset {A}_{0};{A}_{{t}_{1}}\cap {A}_{1}=\varnothing$), which will be completely destructed at some time point. The observed species richness in the intact local community *A*_1_ is thus given by Eq. (1) immediately after habitat loss. However, after a long run since the irreversible loss of ${A}_{{t}_{1}}$, the local community *A*_1_ (in the remaining intact region denoted by ${A}_{0}/{A}_{{t}_{1}}$) will reach a new equilibrium of species richness, which can be estimated as follows:

3 $${S}_{{A}_{0}\backslash {A}_{{t}_{1}}}({A}_{1})=\sum _{n}(1-P(0|n,{A}_{1},{A}_{0}\backslash {A}_{{t}_{1}},\omega ))\varphi (n|{\theta }_{{A}_{0}\backslash {A}_{{t}_{1}}}),$$

where $\varphi (n|{\theta }_{{A}_{0}\backslash {A}_{{t}_{1}}})$ and $P(0|n,{A}_{1},{A}_{0}/{A}_{{t}_{1}},\omega )$ denote the re-fitted SAD and SAAD when the community reaches a new equilibrium after habitat destruction. If SAD, SAAD, or both are shifted to new probability functions with new parameters (these scenarios are also investigated later in this work), it is more appropriate to write the equation as $\varphi ^{\prime} (n|\theta {^{\prime} }_{{A}_{0}\backslash {A}_{{t}_{1}}})$ and $P^{\prime} (0|n,{A}_{1},{A}_{0}/{A}_{{t}_{1}},\omega ^{\prime} )$, respectively. Accordingly, the predicted extinction debt for the local community *A*_1_ in the remaining habitat can be computed as follows:

4 $$G({A}_{1}|{A}_{0},{A}_{{t}_{1}})={S}_{{A}_{0}}({A}_{1})-{S}_{{A}_{0}\backslash {A}_{{t}_{1}}}({A}_{1}).$$

If $G({A}_{1}|{A}_{0},{A}_{{t}_{1}}) > 0$, extinction debt occurs, and if $G({A}_{1}|{A}_{0},{A}_{{t}_{1}}) < 0$, immigration credit occurs. When the area size of the local community is large to include the whole remaining habitat (${A}_{1}={A}_{0}/{A}_{{t}_{1}}$), the above equation actually becomes KH’s original model.

### Delayed loss and gain of endemic species

The loss or gain of the endemic species in the local community *A*_1_ is the part of the magnitude of extinction debt or immigration credit contributed by endemic species that is only found in the community. Based on this definition, their debt or credit magnitude is calculated using Eq. (2) as follows:

5 $$D({A}_{1}|{A}_{0},{A}_{{t}_{1}})={E}_{{A}_{0}}({A}_{1})-{E}_{{A}_{0}\backslash {A}_{{t}_{1}}}({A}_{1}).$$

Again, if $D({A}_{1}|{A}_{0},{A}_{{t}_{1}}) > 0$, extinction debt occurs, and if $D({A}_{1}|{A}_{0},{A}_{{t}_{1}}) < 0$, immigration credit occurs.

### Delayed loss and gain of species with small population sizes

After the loss of ${A}_{{t}_{1}}$, part of the extinction debt or immigration credit derived from species with small population sizes in the local community *A*_1_ can be calculated. For a given small-population threshold *n*_*c*_, the delayed loss or gain of species with population size *n*_*c*_ found in the local community *A*_1_ is calculated as follows:

6 $$\begin{array}{rcl}M({A}_{1}|{A}_{0}),\,{A}_{{t}_{{\rm{l}}}} & = & \sum _{1\le n\le {n}_{c}}(1-P(0|n,{A}_{1},{A}_{0},\omega ))\varphi (n|{\theta }_{{A}_{0}})\\  &  & -\sum _{1\le n\le {n}_{c}}(1-P(0|n,{A}_{1},{A}_{0}\backslash {A}_{{t}_{1}},\omega ))\varphi (n|{\theta }_{{A}_{0}\backslash {A}_{{t}_{1}}}).\end{array}$$

Again, if $M({A}_{1}|{A}_{0},{A}_{{t}_{1}}) > 0$, extinction debt occurs, and if $M({A}_{1}|{A}_{0},{A}_{{t}_{1}}) < 0$, immigration credit occurs.

### Changes in the fraction of unique species for two non-overlapped local communities

Suppose there are two spatially non-overlapped local communities *A*_1_ and *A*_2_ (${A}_{2}\cap {A}_{1}=\varnothing$) in the intact region (${A}_{{t}_{1}}\cap {A}_{1}=\varnothing ;{A}_{{t}_{1}}\cap {A}_{2}=\varnothing$), we can compute the changes in the area-based fraction of unique species number for two non-overlapped local communities after habitat loss as follows:

7 $$\begin{array}{c}{q}_{Jaccard}({A}_{1},\,{A}_{2},\,{A}_{0},\,{A}_{{t}_{{\rm{l}}}}),\\ \quad =\frac{2{S}_{{A}_{0}}({A}_{1}\cup {A}_{2})-{S}_{{A}_{0}}({A}_{2})-{S}_{{A}_{0}}({A}_{1})}{{S}_{{A}_{0}}({A}_{1}\cup {A}_{2})}-\frac{2{S}_{{A}_{0}\backslash {A}_{{t}_{1}}}({A}_{1}\cup {A}_{2})-{S}_{{A}_{0}\backslash {A}_{{t}_{1}}}({A}_{2})-{S}_{{A}_{0}\backslash {A}_{{t}_{1}}}({A}_{1})}{{S}_{{A}_{0}\backslash {A}_{{t}_{1}}}({A}_{1}\cup {A}_{2})}.\end{array}$$

### Two SAD models as demonstrations

#### Canonical lognormal distribution

Suppose the regional SAD for the whole region *A*_0_ is represented by Preston’s canonical lognormal distribution^9, 22^, then $\varphi (n|{{\rm{\Delta }}}_{{A}_{0}})$ is given by

8 $$\varphi (n|{{\rm{\Delta }}}_{{A}_{0}})=\frac{{e}^{{{\rm{\Delta }}}_{{A}_{0}}^{2}}}{n\,\mathrm{ln}\,2}{e}^{-\frac{{(\mathrm{ln}n-2{{\rm{\Delta }}}_{{A}_{0}}^{2})}^{2}}{4{{\rm{\Delta }}}_{{A}_{0}}^{2}}}$$

Correspondingly, either ${\int }_{n=1}^{\exp (4{{\rm{\Delta }}}_{{A}_{0}}^{2})}\varphi (n,{\theta }_{{A}_{0}})={S}_{0}$ or ${\int }_{n=1}^{\exp (4{{\rm{\Delta }}}_{{A}_{0}}^{2})}n\varphi (n,{\theta }_{{A}_{0}})={N}_{0}$ may be used to estimate the parameter ${{\rm{\Delta }}}_{{A}_{0}}$. However, in practice, the latter equality overwhelmingly determines the numerical estimation of the parameter.

Furthermore, in our study, SAAD is supposed to follow finite negative binomial distribution (NBD)^23^ as follows:

9 $$P(m|n,{A}_{1},{A}_{0},k)=\frac{(\begin{array}{c}m+k-1\\ m\end{array})(\begin{array}{c}n-m+k{A}_{0}/{A}_{1}-k-1\\ n-m\end{array})}{(\begin{array}{c}n+k{A}_{0}/{A}_{1}-1\\ n\end{array})},$$

where *k* represents the aggregation degree of species distribution. The above probability model is flexible, because it reaches random distribution as *k* → ∞ and maximal aggregation of species distribution as *k* → 0.

We assess and compare the effects of spatial distribution patterns of species on the estimation of extinction debt and immigration credit. Therefore, we substitute *k* = 1 and *k* = 100 in the above equation to represent distributional aggregation and randomness of species, respectively. We also report the comparative results for two more extreme values *k* = 0.001 and *k* = 1000, representing extreme aggregation and randomness of spatial distribution, respectively.

For example, when species distribution is aggregate (*k* = 1), delayed species loss and gain can be estimated as follows:

10 $$\begin{array}{c}G({A}_{1}|{A}_{0},{A}_{{t}_{1}})={\int }_{n=1}^{\exp (4{{\rm{\Delta }}}_{{A}_{0}}^{2})}(1-P(0|n,{A}_{1},{A}_{0},k=1))\varphi (n,{\theta }_{{A}_{0}})dn\\ \quad \quad \quad \quad \quad \quad \quad -{\int }_{n=1}^{\exp (4{{\rm{\Delta }}}_{{A}_{0}\backslash {A}_{{t}_{1}}}^{2})}(1-P(0|n,{A}_{1},{A}_{0}\backslash {A}_{{t}_{1}},k=1))\varphi (n,{\theta }_{{A}_{0}\backslash {A}_{{t}_{1}}})dn,\end{array}$$

where ${{\rm{\Delta }}}_{{A}_{0}\backslash {A}_{{t}_{1}}}$ is numerically determined by the following equation,

11 $${\int }_{n=1}^{\exp (4{{\rm{\Delta }}}_{{A}_{0}\backslash {A}_{{t}_{1}}}^{2})}n\varphi (n|{\theta }_{{A}_{0}\backslash {A}_{{t}_{1}}})dn={N}_{{A}_{0}\backslash {A}_{{t}_{1}}}=(1-{A}_{{t}_{1}}/{A}_{0}){N}_{0}.$$

The last equality in Eq. (11) is conditioned on the fact that the total number of individuals species is proportional to the area size, which is a widely applied prerequisite in many macro-ecological studies^9, 24, 25^.

For lognormal SAD model, KH presented an asymptotic formula for estimating species number after the community reaches a new equilibrium since the point of habitat destruction as ${S}_{{A}_{0}\backslash {A}_{{t}_{1}}}({A}_{1})={S}_{0}{({A}_{1}/{A}_{0})}^{0.25}$. This is a classic power-law species-area relation with a widely used slope 0.25 for a log transformation. This formula may be used for large Δ, but for guaranteeing computational accuracy, we numerically estimate the parameter Δ in $\varphi (n|{\rm{\Delta }})$.

#### Single-parameter logseries distribution

Fisher’s logseries distribution was a widely used SAD model in ecology, because it can predict a high volume of rare species in the ecological community, which has a form as follows^26^:

12a $$\varphi (n|\alpha ,x)=\alpha \frac{{x}^{n}}{n}.$$

However, because it contains two unknown parameters *α* and *x*, it is not suitable for estimating the number of species after habitat loss based on one observation on the number of individuals. Whereas, it is possible if one parameter (e.g. *α* here) is assumed to be constant before and after habitat loss and the estimation of species number after habitat loss can be achieved, as in KH’s study. Based on this assumption, the loss of species richness is proportional to the habitat loss.

The same issue is applied to other variant logseries models, such as the one derived from the maximum entropy theory^24, 27, 28^, which is given by

12b $$\varphi (n|{\lambda }_{{A}_{0}})=\frac{{N}_{0}}{{S}_{0}}\frac{(1-{e}^{-{\lambda }_{{A}_{0}}})}{({e}^{-{\lambda }_{{A}_{0}}}-{e}^{-{\lambda }_{{A}_{0}}({N}_{0}+1)})}\times {e}^{-{\lambda }_{{A}_{0}}n}/n.$$

When *N*_0_ goes to infinity and *N*_0_/*S*_0_ approaches to a constant, Eq. (12b) becomes very similar to Fisher’s original version (Eq. 12a). Since *N*_0_/*S*_0_ can be observed, Eq. (12b) contains only one unknown parameter ${\lambda }_{{A}_{0}}$, which can be estimated before habitat loss. However, after habitat loss, Eq. (12b) would contain two unknown parameters actually (${S}_{{A}_{0}\backslash {A}_{{t}_{1}}}({A}_{1})$ and ${\lambda }_{{A}_{0}\backslash {A}_{{t}_{1}}}$), but we only have the number of individuals after habitat loss in hand. Thus, it is not directly applicable to the present model unless some assumptions have been made. For example, like the discussion above, the parameter ${\lambda }_{{A}_{0}}$ can be assumed to be constant (so ${\lambda }_{{A}_{0}\backslash {A}_{{t}_{1}}}={\lambda }_{{A}_{0}}$) or linearly related to the percent of lost habitat.

To break through the limit, a promising way is to introduce another variant logseries model containing only a single unknown parameter. Thus, it is applicable to predict the number of species after habitat loss. It is possible to extend the logseries model developed by R.A. Fisher, in which he refers the original model to another widely used probability model in ecology, namely the ordinary NBD. The ordinary NBD actually was used in modelling species abundance and later introduced to describe species distributional patterns (aggregation, random, or regular)^23, 26, 29^. However, ordinary NBD-driven logseries model (Eq. 12a) was not a proper probability model and degenerate or undefined at the zero point. To overcome this problem, we introduced a truncated NBD model, and used that to induce a single-parameter logseries model as follows:

12c $${\varphi }^{1}(n|{\gamma }_{{A}_{0}})={C}_{1}\frac{{\gamma }_{{A}_{0}}}{n}{(1-{e}^{-1/{\gamma }_{{A}_{0}}})}^{n},n=1,\mathrm{2...}{N}_{0},$$

where ${\gamma }_{{A}_{0}}$ is the unknown parameter, and *C*_1_ is a normalisation factor to make $\sum _{i=1}^{{N}_{0}}{\varphi }^{1}(n|{\gamma }_{{A}_{0}})=1$. Details of the derivation of this model could be found in the Supplementary information. However, we did not fit ${\gamma }_{{A}_{0}}$ directly; instead, as shown in the Supplementary information, it is easy to show that it is area-dependent, i.e. ${\gamma }_{{A}_{0}}={[\mathrm{ln}(1+{A}_{0}/\omega )]}^{-1}$, in which *ω* is a hyper-parameter that is assumed to be a constant.

The parameter ${\gamma }_{{A}_{0}}$ (actually we estimate the hyper-parameter *ω* here) can be numerically estimated by solving the following equality

13 $$\sum _{n=1}^{{N}_{0}}n\times {\varphi }^{1}(n|{\gamma }_{{A}_{0}})=E[n]\approx \frac{{N}_{0}}{{S}_{0}}.$$

After habitat destruction, ${\gamma }_{{A}_{0}}$ and *N*_0_ in Eq. (13) are replaced by ${\gamma }_{{A}_{0}\backslash {A}_{{t}_{1}}}$ and $\frac{{A}_{0}-{A}_{{t}_{1}}}{{A}_{0}}{N}_{0}$, respectively. Here, ${\gamma }_{{A}_{0}\backslash {A}_{{t}_{1}}}={[{\rm{l}}{\rm{n}}(1+({A}_{0}-{A}_{{t}_{1}})/\omega )]}^{-1}$. As a result, the new species number at equilibrium after habitat loss can be estimated from Eq. (13). Apparently, the parameters ${\gamma }_{{A}_{0}}$ and ${\gamma }_{{A}_{0}\backslash {A}_{{t}_{1}}}$ are unequal (though the hyper-parameter *ω* is fixed), strictly following our relaxed assumption that parameters can vary before and after habitat loss.

In our study, we utilised the above two regional SADs (Eqs 8 and 12c) and the SAAD model, which follows the finite negative binomial distribution, to demonstrate the effects of habitat destruction on local intact communities in the remaining habitats. We specifically investigated the effects of habitat loss on the delayed species gain or loss of a single local community and tested whether endemic and rare species may contribute to the gain or loss of species. We also investigated the effects of habitat loss on the alteration of community structure (quantified as the changes in the fraction of unique species between two random local communities). Moreover, we tested the contribution of endemic or small-population species to the loss and gain of biodiversity. For these analyses, we compared two distinct forms of species distribution, namely randomness (*k* = 100 and 1000, respectively) and aggregation (*k* = 1 and 0.001, respectively). We assessed whether shifting species distribution pattern (difference in distributional aggregation or randomness degree of species before and after habitat destruction) result in new species and community dynamic patterns in local communities.

## Results

### Delayed biodiversity loss and biodiversity accrual in a single local community

**Figure 2:**
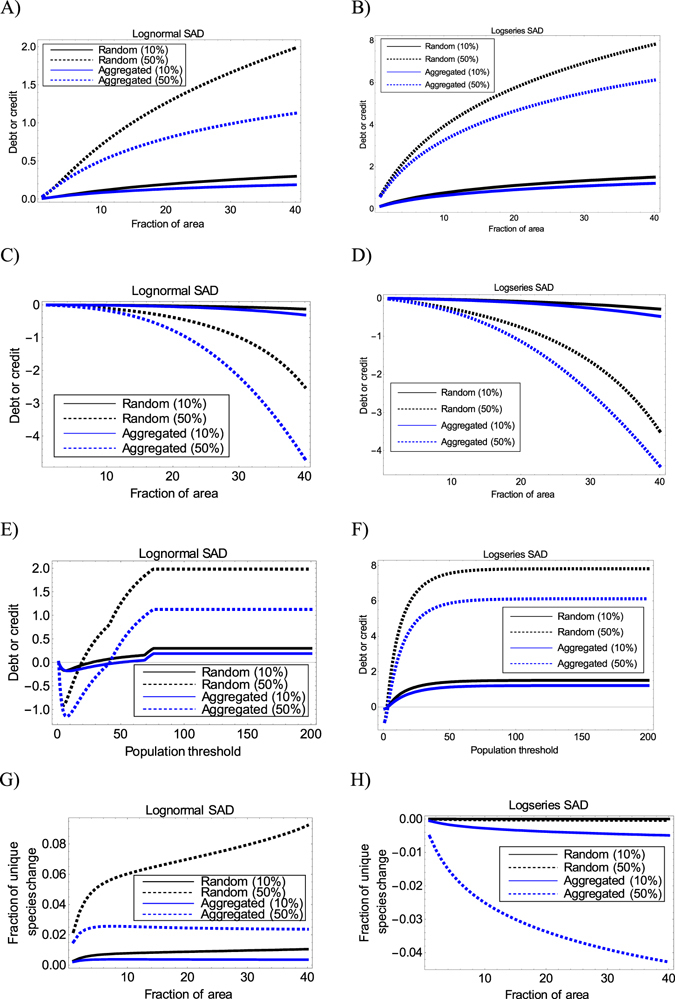
Prediction of extinction debt or immigration credit for a single local community and changes in the fraction of unique species for a pair of local communities in the remaining intact region. All scenarios here assume that both SAD and SAAD are fixed before and after habitat loss. Species distribution is assumed to be random (*k* = 100) or aggregate (*k* = 1). (**A**,**B**) Extinction debt or immigration credit as a function of the area size of a single local community; (**C**,**D**) extinction debt or immigration credit for endemic species in a single local community; (**E**,**F**) extinction debt or immigration credit contributed by small-population species (the area of the local community is fixed to have a fraction 0.4 over the region); and (**G**,**H**) changes in the fraction of unique species as a function of the area size of one local community in a pair (the area size of the other community is fixed to be 10% of the whole region). Before habitat destruction, the region was assumed to have 30 species and 200 individuals in the model. In all the subplots, dashed lines represent the outcomes when 50% of the region is destructed, while solid lines represent the outcomes when only 10% of the region is destructed.

Regardless of the regional SAD and SAAD models being used (as long as they are always fixed before and after habitat loss), an intact local community *A*_1_ with a larger area size would have a higher magnitude of extinction debt for a given percent of habitat loss (Fig. 2A,B). For both SADs, an increase in the habitat loss would lead to higher extinction debts in the intact local community (Fig. 2A,B). Moreover, the debt becomes higher when species distribution is less aggregate (Fig. 2A,B).

Interestingly, no extinction debts for endemic species are observed in both the SAD models (Fig. 2C,D). More endemic species are found in the local community (thus immigration credit) when species distribution becomes aggregate and habitat destruction degree is high (Fig. 2C,D). By contrast, for species with different population sizes (not necessarily endemic), when the population threshold is small and only rare species are included, the immigration credit is observed in both the SAD models (Fig. 2E,F). When more species with larger or intermediate population sizes are taken into account (by setting a higher population threshold), immigration credits disappear, while extinction debts emerge, regardless of species distribution pattern and habitat loss degree (Fig. 2E,F).

### Delayed changes in the fraction of unique species for two local communities

There are some interesting patterns for the changes in the fraction of unique species between two local communities. Despite species distribution status and habitat loss degree, the fraction of unique species between two non-overlapped local communities would decrease for the lognormal model and increase for the logseries model after habitat loss. In other words, the changes in the fraction of unique species number between two non-overlapped local communities have positive and negative values as shown in Fig. 2G,H, respectively. For the lognormal model, the reduction in the fraction of unique species becomes larger when the area size of a local community in a pair increases (the size of the other community is fixed) or when the habitat destruction degree is higher (Fig. 2G). By contrast, for the logseries model, the magnitude of change in the fraction of unique species of two non-overlapped local communities would always increase when the area size of either local community increases (Fig. 2H). Moreover, when species distribution is random, there is almost no change on the fraction of unique species for the logseries model, no matter how the habitat loss degree is (Fig. 2H).

### Effects of shifting SAAD

**Figure 3:**
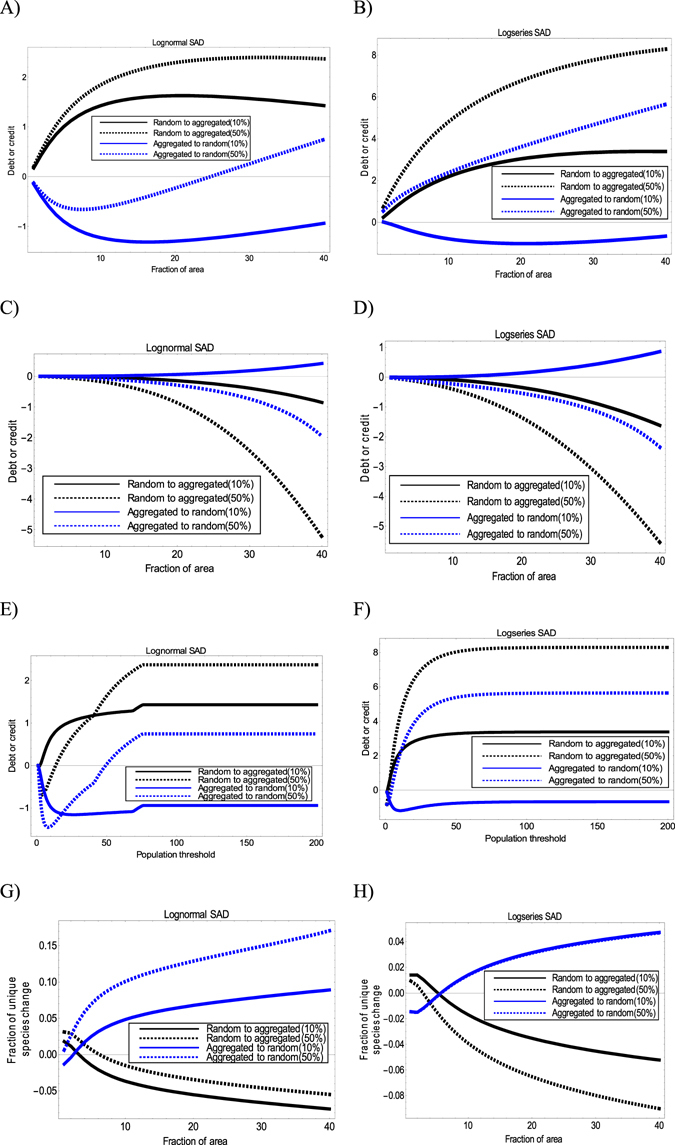
Prediction of extinction debt or immigration credit for a single local community and changes in the fraction of unique species for a pair of local communities in the remaining intact region. All scenarios here assume SAD to be fixed, while SAAD can shift (exchangeable between random and aggregated distributions) before and after habitat loss. Species distribution is assumed to be random (*k* = 100) or aggregate (*k* = 1). (**A**,**B**) Extinction debt or immigration credit as a function of the area size of a single local community; (**C**,**D**) extinction debt or immigration credit for endemic species in a single local community; (**E**,**F**) extinction debt or immigration credit contributed by small-population species (the area of the local community is fixed to have a fraction 0.4 over the region); and (**G**,**H**) changes in the fraction of unique species as a function of the area size of one local community in a pair (the area size of the other community is fixed to be 10% of the whole region). Before habitat destruction, the region is assumed to have 30 species and 200 individuals in the model. In all the subplots, dashed lines represent the outcomes when 50% of the region is destructed, while solid lines represent the outcomes when only 10% of the region is destructed.

When species distribution pattern is allowed to vary before and after habitat loss, both consistent and new patterns are observed (Fig. 3). The consistent aspects include immigration credit patterns for endemic species of a single local community (Fig. 3C,D), which are similar to those results when SAAD is fixed (Fig. 2C,D). The contribution of species with different population sizes to the overall debt or credit magnitude of the whole community is similar (Figs 2E,F versus 3E,F).

Other than these, some new interesting patterns emerge. Firstly, immigration credit can occur in the local community when species distribution shifts from aggregation to randomness (Fig. 3A,B). Moreover, when habitat destruction degree is low, immigration credit always persists, no matter how the area size of the local community varies (Fig. 3A,B). Secondly, the fraction of unique species can decrease initially when the local community size is small but increase later when SAAD is shifted from randomness to aggregation (Fig. 3G,H). Thirdly, irrespective of the population threshold set and the SAD model used, immigration credit persists when spatial distribution is shifted from aggregation to randomness and habitat loss percent is 10% (Fig. 3E,F). Finally, immigration credits may occur for endemic species in both SAD models, which were not observed when SAAD was fixed (Figs 2C,D versus 3C,D).

### Effects of extreme species distribution

Most results for the extreme aggregation (*k* = 0.001) and randomness (*k* = 1000) scenarios (Supplementary Fig. S1) are similar to those reported above when *k* = 1 and *k* = 100 are assumed (Fig. 2 versus Supplementary Fig. S1). Moreover, akin to the random distribution case in the logseries model (Fig. 2H), no (or nearly no) changes in the fraction of unique species have been found in the extreme aggregation case (*k* = 0.001), regardless of the SADs used (Supplementary Fig. S1G,H).

## Discussion

The present study develops a very flexible framework to predict the delayed responses of species diversity and community structure due to habitat destruction in local communities with various area sizes. The results provide primary theoretical evidence to demonstrate how extinction debt or immigration credit emerges; how the fraction of unique species changes; and how they vary along with changing area size of the local community, changing levels of habitat destruction, shifting species distribution, and shifting regional species abundance. In particular, this study is the first to explicitly demonstrate that habitat destruction can result in more endemic or rare species. Correspondingly, endemic and rare species do not necessarily contribute to extinction debt. Instead, they can contribute to immigration credit. Our study proves that there will be no change in the fraction of unique species when species distribution is highly aggregate.

The present results provide some interesting patterns. For example, for the single-parameter logseries SAD model, extinction debts can occur in local intact communities when habitat loss happens elsewhere (Fig. 3B). For the lognormal model, extinction debt is expected when species distribution is random (Figs 2A and 3A)^9^, but immigration credit can also occur as long as SAAD can change (Fig. 3A and B). These findings suggest that there are diverse delayed responses of ecological communities, depending on a variety of ecological factors (Figs 2 and 3 and Supplementary Figs S1 and S2). More importantly, these diverse area-dependent change in local diversity and community structure predicted by the general framework perfectly fits the possible empirical observations, such as climate change or habitat loss that may increase or decrease alpha diversity, the fraction of unique species, or both (Table 1).

**Table 1:** Explaining diverse empirical patterns on the local-regional diversity change and changes in the fraction of unique species caused by habitat destruction (or climate change) using the general framework proposed in the present study.

Possible empirical patterns		Possible explanations using the proposed framework
Local diversity versus regional diversity change		
1.	Local diversity increases; Regional diversity increases	Immigration credit occurs across all the spatial scales. This can happen when SAAD is shifted from aggregation to randomness and habitat destruction is low (Fig. 3A,B).
2.	Local diversity increases; Regional diversity decreases	Immigration credit occurs at local communities with small area sizes, while extinction debt occurs for the whole remaining region. This may happen when SAD is fixed to be lognormal, while SAAD is allowed to shift from aggregation to randomness and when habitat destruction is high (Fig. 3A).
3.	Local diversity decreases; Regional diversity decreases	Extinction debt occurs at all spatial scales. This may happen when (1) both SAD and SAAD are fixed before and after habitat loss (Fig. 2A,B) or (2) when SAD is fixed, while SAAD is shifted from randomness to aggregation (Fig. 3A,B).
Changes in the fraction of unique species		
1.	Fraction of unique species increases after habitat loss	This may occur (1) when the sum of area sizes of a pair of local communities is large enough and when SAAD is shifted from randomness to aggregation, while SAD is fixed (Fig. 3G,H) or (2) both SAD and SAAD are fixed in logseries model (Fig. 2H).
2.	Fraction of unique species decreases after habitat loss	This may occur (1) when both SAD and SAAD are fixed in lognormal model (Fig. 2G) or (2) when SAAD is shifted from aggregation to randomness, while SAD is fixed (Fig. 3G,H).
3.	No fraction of unique species change after habitat loss	This may occur when species distribution is highly aggregate, regardless of SAD models used (Supplementary Figs. S1G,H).
Alpha diversity versus change in the fraction of unique species		
1.	Local alpha diversity increases; fraction of unique species increases	This may occur when species distribution pattern is extremely aggregated in lognormal model (Fig, S1A versus S1G).
2.	Local alpha diversity increases; fraction of unique species decreases	This may occur when SAD is fixed, while SAAD is shifted from aggregation to randomness (Fig. 3A versus 3G or 3B versus 3H).
3.	Local alpha diversity decreases; fraction of unique species decreases	This may occur when both SAD and SAAD are fixed (Fig. 2A versus 2G or 2B versus 2H).
4.	Local alpha diversity decreases; fraction of unique species increases	This may occur when SAAD is shifted from randomness to aggregation (Fig. 3A versus 3G or 3B versus 3H).

Contradictory to the argument made in KH’s paper, the present study theoretically demonstrates that logseries SAD can predict either extinction debt or immigration credit in the local community (Figs 2B and 3B, and Supplementary Fig. S1B). In particular, for the most restricted case when both SAD and SAAD are fixed (which was investigated in KH’s study), extinction debt occurs (Fig. 2B and Supplementary Fig. S1B). So how can KH show that logseries model predicts zero extinction debt in their study? A key reason is that their study assumed the alpha parameter to be constant when applying Fisher’s logseries model (Eq. 12a). Finally, it is worth mentioning that, in another study^30^, Fisher’s logseries model actually has been employed to predict delayed extinction of Amazonian trees when equipped with a neutral model that they developed in their study. As such, the present findings and the previous study^30^ jointly support the fact that logseries SAD model shall predict extinction debts, depending on factors like model formulation method.

An interesting finding of this study is that endemic species (subplots C and D in Figs 2, 3 and Supplementary Fig. S1) or species with small population sizes (subplots E and F in Figs 2, 3 and Supplementary Fig. S1) are predicted to increase in local communities in many cases. Thus, immigration credit is further strengthened, particularly for endemic species, when habitat destruction degree is high (subplots C and D in Figs 2, 3 and Supplementary Fig. S1). One possible reason is that habitat destruction drives many common species to go extinct and become rare or endemic species (because some of their populations in other areas have gone extinct), resulting in a higher degree of endemism and rarity in local communities in the remaining habitat after destruction. This evidence is supported by high extinction debts in non-endemic species in the focused local community for many scenarios (Supplementary Fig. S2). This can happen when the whole community has a high debt (Fig. 2A,B), while endemic species have an immigration credit (Fig. 2C,D). The subtraction of both will lead to a great loss or small gain in non-endemic species after habitat destruction (Supplementary Fig. S2). Greater loss of non-endemic species is found when the sampling area becomes larger (Supplementary Fig. S2). This implied that common species may be more vulnerable to habitat destruction and may undergo delayed extinction. This can be reasonable, if these common species prefer to inhabit the destructed habitats, or the destructed habitats become keystone ecosystems to support all species.

Habitat destruction degree, area size of the local community, SAD, and SAAD models jointly play deterministic roles affecting the delayed changes in local diversity and community structure. In particular, shifting SAAD and extreme species distribution can generate new patterns (Fig. 3 and Supplementary Fig. S1). For example, under extreme aggregation condition (*k* = 0.001), there is no net change in the fraction of unique species (Supplementary Fig. S1G,H). The key fact is that the fraction of unique species computed for a pair of local communities under maximal aggregation condition is one, regardless of regional SADs and habitat destruction degree used (see the proof of Theorem 2 in the Supplementary Information). Thus, these results imply that under extreme aggregation condition, each local community would be predicted to have completely different species composition (and all of them are endemic) with respect to any other communities.

It is worth mentioning that zero change on the fraction of unique species does not merely occur in the extreme aggregation condition. Actually, when species distribution is very random, the fraction of unique species in the single-parameter logseries SAD model is also unchanged, regardless of habitat loss degree (Fig. 2H). The reason is greatly due to the extreme right-skewed curve shape of the logseries SAD model (thus predicting rare species everywhere) and the asymptotic binomial distribution pattern of species when *k* is large (see the proof of Theorem 3 in the Supplementary Information). Finally, in the proof of Theorem 3, it can be seen that the change on the fraction of unique species is not related to the area size of the lost habitat after some algebra. This well explains why the zero net change occurs as long as species distribution is random.

In conclusion, the present study has theoretically discovered very diverse and interesting post-destruction responses of species diversity and community structure, which can match and explain a variety of empirical observations (Table 1). We have shown that there are area- and distribution-dependent transitions between extinction debts and immigration credits. Endemic species increase in the local community after habitat loss in many situations (Fig. 2C,D and Supplementary Fig. S1C,D). The fraction of unique species may increase, decrease, or remain unchanged depending on multiple factors, including the SAD model used, the area size of the local community, habitat destruction, and species distribution pattern (Figs 2G,H and 3G,H and Supplementary Fig. S1G,H). There is no change in the fraction of unique species number in extremely aggregate distribution situation (*k* → 0), regardless of regional SAD models used (Supplementary Fig. S1G,H).

For future implications, the succeeding steps are to further verify these diverse theoretical patterns predicted by the proposed framework at local-scale field experiments. Moreover, because there have been some empirical studies reporting the evidence of extinction debts^6, 31^, data collected from previous studies (if available) might be re-used to examine the possible alteration of community structure and change in the fraction of unique species after habitat loss. In addition, there are many other intriguing areas open for future research. For example, if observational data from the fields are available, it will be interesting to verify whether the fitted SAD models before and after habitat loss would have the same parametric form. It is also interesting to experimentally test the alteration in species distribution after habitat loss and its influence on the local community structure.

## Electronic supplementary material

Supplementary Information

## References

[1] Tilman D, May R, Lehman C, Nowak M (1994). Habitat destruction and the extinction debt. Nature.

[2] Isbell F, Tilman D, Polasky S, Loreau M (2015). The biodiversity-dependent ecosystem service debt. Ecol. Lett..

[3] Jackson S, Sax D (2010). Balancing biodiversity in a changing environment: extinction debt, immigration credit and species turnover. Trends Ecol. Evol..

[4] Kuussaari M (2009). Extinction debt: a challenge for biodiversity conservation. Trends Ecol. Evol..

[5] Stork N (2010). Re-assessing current extinction rates. Biodivers. Conserv..

[6] Helm A, Hanski I, Partel M (2006). Slow response of plant species richness to habitat loss and fragmentation. Ecol. Lett..

[7] Dullinger S (2012). Extinction debt of high-mountain plants under twenty-first-century climate change. Nat. Clim. Chang..

[8] Cowlishaw G (1999). Predicting the pattern of decline of African primate diversity: an extinction debt from historical deforestation. Conserv. Biol..

[9] Kitzes J, Harte J (2015). Predicting extinction debt from community patterns. Ecology.

[10] Diamond J (1972). Biogeographic kinetics: estimation of relaxation times for avifaunas of southwest Pacific islands. PNAS.

[11] Halley J, Iwasa Y (2011). Neutral theory as a predictor of avifaunal extinctions after habitat loss. PNAS.

[12] Wearn O, Reuman D, Ewers R (2012). Extinction debt and windows of conservation opportunity in the Brazilian Amazon. Science (80)..

[13] Gonzalez A (2016). Estimating local biodiversity change: a critique of papers claiming no net loss of local diversity. Ecology.

[14] Sax D, Gaines S (2003). Species diversity: from global decrease to local increases. Trends Ecol. Evol..

[15] Vellend M (2013). Global meta-analysis reveals no net change in local-scale plant biodiversity over time. PNAS.

[16] Socolar J, Gilroy J, Kunin W, Edwards D (2016). How should beta-diversity inform biodiversity conservation?. Trends Ecol. Evol..

[17] Knop E (2016). Biotic homogenization of three insect groups due to urbanization. Glob. Chang. Biol..

[18] Magurran A (2016). How ecosystems change. Science (80)..

[19] Dornelas M (2014). Assemblage time series reveal biodiversity change but not systematic loss. Science (80-)..

[20] Wilber, M., Kitzes, J. & Harte, J. Scale collapse and the emergence of the power law species-area relationship. *Glob*. *Ecol*. *Biogeogr*, doi:10.1111/geb.12309 (2015).

[21] Zillio T, He F (2010). Inferring species abundance distribution across spatial scales. Oikos.

[22] Preston F (1962). The canonical distribution of commonness and rarity: part I. Ecology.

[23] Zillio T, He F (2010). Modeling spatial aggregation of finite populations. Ecology.

[24] Harte J, Smith A, Storch D (2009). Biodiversity scales from plots to biomes with a universal species-area curve. Ecol. Lett..

[25] Colwell R (2012). Models and estimators linking individual-based and sample-based rarefaction, extrapolation and comparison of assemblages. J. Plant Ecol..

[26] Fisher R, Corbet A, Williams C (1943). The relation between the number of species and the number of individuals in a random sample of an animal population. J. Anim. Ecol..

[27] Harte J, Zillio T, Conlisk E, Smith A (2008). Maximum entropy and the state variable approach to macroecology. Ecology.

[28] Harte, J. *Maximum entropy and ecology*. (Oxford University Press, Oxford, 2011).

[29] Brown J, Mehlman D, Stevens G (1995). Spatial variation in abundance. Ecology.

[30] Gilbert B, Laurance W, Leigh E, Nascimento H (2006). Can neutral theory predict changes in Amazonian forest fragments?. Am. Nat..

[31] Highland S, Jones J (2014). Extinction debt in naturally contracting moutnain meadows in the Pacific Northwest, USA: varying responses of plants and feeding guilds of nocturnal moths. Biodivers. Conserv..

