# Supplementary material for: A general framework for predicting delayed responses of ecological communities to habitat loss
Source: Sci Rep. 2017 Apr 20;7:998. doi: 10.1038/s41598-017-01070-2 (PMC5430564; doi:10.1038/s41598-017-01070-2)
Supplement: Supplementary file 1 — Supplementary Information [file 41598_2017_1070_MOESM1_ESM.doc]

**Supplementary Information:**

**A general framework for predicting delayed responses of ecological communities to habitat loss**

Youhua Chen^1, 3*^, Tsung-Jen Shen^2*^

1, Department of Renewable Resources, University of Alberta, Edmonton, T6G 2H1, Canada

2, Institute of Statistics & Department of Applied Mathematics, National Chung Hsing University, 250 Kuo Kuang Road, Taichung 40227 Taiwan, ROC

3, School of Health Sciences, Wuhan University, Wuhan 430072, China

*Emails for correspondence: haydi@126.com; tjshen@nchu.edu.tw

**A single-parameter logseries model derived from a truncated NBD model**

To derive a single-parameter logseries model, suppose that an ecological community is studied within a finite area denoted by *A*, we consider a truncated NBD that takes the area size of the studied region into account in comparison to the ordinary NBD. The probability function of this truncated NBD is given by,

(S1)

where $C_{0}=\left(1-\left(\frac{\omega}{\omega+A}\right)^{\kappa}\right)^{-1}$ and $\omega$ and $\kappa$ are two unknown parameters. The truncation at *zero* can allow the derived logeseries model to be a proper probability function and have a single unknown parameter. To show these, using this model with $\kappa\to0$, the limiting distribution (please see the detailed proof of Thereom 1 below) can be derived by

**, (S2)**

where let $x_{A_{0}}=A_{0}/(\omega+A_{0})$ and $\gamma_{A_{0}}=\left[\ln(1+A_{0}/\omega)\right]^{-1}$ which respectively corresponds to *x* and $\alpha$ in Fisher’s logseries distribution (i.e., Eq. 12 in the main text). As a result, when relating $x_{A_{0}}$ to $\gamma_{A_{0}}$ here, the single-parameter logseries distribution can be concisely expressed as,

. **(S3)**

However, because our study focuses on a finite population size of species, we further truncate the above model Eq. (S3) to exclude those probabilities that are not part of the population size of the species $N_{0}$ as

**, (S4)**

where  is a normalisation or truncation factor. Note that the proposed model (Eq. S4 or Eq. 12 in the main text) is of only one parameter $\gamma_{A_{0}}$ (actually the hyper-parameter $\omega$), whereas Fisher’s logseries distribution has two parameters *x* and $\alpha$ (as in Eq. 12 in the main text). Because the curve shape of the logseries model is extremely right-skewed, numerical results can show the normalisation factor $C_{1}$ is very close to 1. So, in practice, Eq. (S3) was used for deriving Theorem 3 below (because when $C_{1}$=1, Eq. S4 is identical to S3).

**Theorem 1: The truncated NBD (Eq. S1), when** $\kappa\to0$**, has the same probability function form as the original Fisher’s logseries model (Eq. S3).**

Proof:

First, we write the probability function of the truncated NBD as the following form

Accordingly, using the tractable expression above, we can show

**Theorem 2: No change of fraction of unique species for a pair of local communities after habitat loss when species distribution is highly aggregate ($k\to0$)**

**Proof:**

The finite NBD (Zillio & He 2010) is given by,

$$P(m|n,A_{1},A_{0},k)=\frac{\left(\begin{matrix} m+k-1 \\ m \end{matrix}\right)\left(\begin{matrix} n-m+kA_{0}/A_{1}-k-1 \\ n-m \end{matrix}\right)}{\left(\begin{matrix} n+kA_{0}/A_{1}-1 \\ n \end{matrix}\right)}$$

For the maximal aggregation of species distribution, Kitzes and Harte (2015, p.2131) mentioned,

$\lim_{k\to0} P(0|n,A_{1},A_{0},k)=1-A_{1}/A_{0}$ **(S5)**

Therefore, for any regional SAD model , the predicted species richness for the local community before and after habitat loss is as follows:

**(S6)**

Specifically, the fractions of unique species number before and after habitat loss are respectively given by

**(S7)**

Consequently, the change of fraction of unique species number after habitat loss is zero ($q_{before}-q_{after}=0$), a constant, which is not related to the changing area size of the local community.

**Theorem 3: For the logseries model in Eq. (S4), almost no change of fraction of unique species number for a pair of local communities after habitat loss, when species distribution is highly random ($k\to\infty$).**

**Proof:**

We firstly show that $\lim_{\kappa\to\infty} P(0|n,A_{1},A_{0},k)=\left(1-\frac{A_{1}}{A_{0}}\right)^{n}$, though the result was implicitly revealed by Kitzes and Harte (2015, p.2131). Note that

$\begin{matrix} P(0|n,A_{1},A_{0},k)=\frac{\left(\begin{matrix} n+kA_{0}/A_{1}-k-1 \\ n \end{matrix}\right)}{\left(\begin{matrix} n+kA_{0}/A_{1}-1 \\ n \end{matrix}\right)}=\frac{\left(\begin{matrix} n+k(A_{0}/A_{1}-1)-1 \\ n \end{matrix}\right)}{\left(\begin{matrix} n+kA_{0}/A_{1}-1 \\ n \end{matrix}\right)} \\ =\prod_{j=1}^{n} \left[\frac{n+k\left(A_{0}/A_{1}-1\right)-j}{n+k\left(A_{0}/A_{1}\right)-j}\right] \end{matrix}$**, (S8)**

and moreover

$\lim_{\kappa\to\infty} \frac{n+k\left(A_{0}/A_{1}-1\right)-j}{n+k\left(A_{0}/A_{1}\right)-j}=1-\frac{A_{1}}{A_{0}}$, **(S9)**

thus we have the result. Based on the expression in Eq. (S8), when $k\to0$, one can get the result as in Eq. (S5) as well.

Let$g_{A_{0}}(A_{1})=1-A_{1}/A_{0}$, and $\eta(A)=\omega/\left(A+\omega\right)$, thus we have

, **(S10)**

And similarly,

**(S11)**

Note that, as mentioned earlier, to simplify the derivation, approximations in Eqs. (S10) and (S11) are used by ignoring the normalisation factor in Eq. (S4). Moreover, since the logseries model strictly decays very fast, the summation from 1 to a finite value (e.g., *N_0_*) can be well approximated by the summation from 1 to infinity, which has a very simple closed form.

As a result, the fractions of unique species number before and after habitat loss are respectively given as follows:

$$q_{before}=\frac{2S_{A_{0}}(A_{1}\cup A_{2})-S_{A_{0}}(A_{2})-S_{A_{0}}(A_{1})}{S_{A_{0}}(A_{1}\cup A_{2})}=2-\frac{S_{A_{0}}(A_{2})}{S_{A_{0}}(A_{1}\cup A_{2})}-\frac{S_{A_{0}}(A_{1})}{S_{A_{0}}(A_{1}\cup A_{2})}$$

and

$q_{after}=\frac{2S_{A_{0}\backslash A_{t_{1}}}(A_{1}\cup A_{2})-S_{A_{0}\backslash A_{t_{1}}}(A_{2})-S_{A_{0}\backslash A_{t_{1}}}(A_{1})}{S_{A_{0}\backslash A_{t_{1}}}(A_{1}\cup A_{2})}=2-\frac{S_{A_{0}\backslash A_{t_{1}}}(A_{2})}{S_{A_{0}\backslash A_{t_{1}}}(A_{1}\cup A_{2})}-\frac{S_{A_{0}\backslash A_{t_{1}}}(A_{1})}{S_{A_{0}\backslash A_{t_{1}}}(A_{1}\cup A_{2})}$.

Therefore,

$q_{before}-q_{after}=\left(\frac{S_{A_{0}\backslash A_{t_{1}}}(A_{2})}{S_{A_{0}\backslash A_{t_{1}}}(A_{1}\cup A_{2})}-\frac{S_{A_{0}}(A_{2})}{S_{A_{0}}(A_{1}\cup A_{2})}\right)+\left(\frac{S_{A_{0}\backslash A_{t_{1}}}(A_{1})}{S_{A_{0}\backslash A_{t_{1}}}(A_{1}\cup A_{2})}-\frac{S_{A_{0}}(A_{1})}{S_{A_{0}}(A_{1}\cup A_{2})}\right)$.

Specifically, we derive the difference from two terms below

$\begin{matrix} \frac{S_{A_{0}}(A_{1})}{S_{A_{0}}(A_{1}\cup A_{2})}-\frac{S_{A_{0}\backslash A_{t_{1}}}(A_{1})}{S_{A_{0}\backslash A_{t_{1}}}(A_{1}\cup A_{2})} \\ \approx\frac{\left[1-\frac{\ln\left(1-g_{A_{0}}(A_{1})\eta(A_{0})\right)}{\ln\left(1-\eta(A_{0})\right)}\right]}{1-\frac{\ln\left(1-g_{A_{0}}(A_{1}+A_{2})\eta(A_{0})\right)}{\ln\left(1-\eta(A_{0})\right)}}-\frac{\left[1-\frac{\ln\left(1-g_{A_{0}\backslash A_{t_{1}}}(A_{1})\eta(A_{0}-A_{t_{1}})\right)}{\ln\left(1-\eta(A_{0}-A_{t_{1}})\right)}\right]}{1-\frac{\ln\left(1-g_{A_{0}\backslash A_{t_{1}}}(A_{1}+A_{2})\eta(A_{0}-A_{t_{1}})\right)}{\ln\left(1-\eta(A_{0}-A_{t_{1}})\right)}} \\ \approx\frac{\eta(A_{0})-g_{A_{0}}(A_{1})\eta(A_{0})}{\eta(A_{0})-g_{A_{0}}(A_{1}+A_{2})\eta(A_{0})}-\frac{\eta(A_{0}-A_{t_{1}})-g_{A_{0}\backslash A_{t_{1}}}(A_{1})\eta(A_{0}-A_{t_{1}})}{\eta(A_{0}-A_{t_{1}})-g_{A_{0}\backslash A_{t_{1}}}(A_{1}+A_{2})\eta(A_{0}-A_{t_{1}})} \\ =\frac{1-g_{A_{0}}(A_{1})}{1-g_{A_{0}}(A_{1}+A_{2})}-\frac{1-g_{A_{0}\backslash A_{t_{1}}}(A_{1})}{1-g_{A_{0}\backslash A_{t_{1}}}(A_{1}+A_{2})} \\ =\frac{A_{1}}{A_{1}+A_{2}}-\frac{A_{1}}{A_{1}+A_{2}}=0 \end{matrix}$ **(S12)**

and similarly

$\frac{S_{A_{0}}(A_{2})}{S_{A_{0}}(A_{1}\cup A_{2})}-\frac{S_{A_{0}\backslash A_{t_{1}}}(A_{2})}{S_{A_{0}\backslash A_{t_{1}}}(A_{1}\cup A_{2})}\approx0$, **(S13)**

where approximations in Eqs. (S12) and (S13) are based on $\ln(1+x)\approx x$ (Taylor’s expansion) when *x* is small. Such approximations are valid because terms like$g_{A_{0}}(A_{1}+A_{2})\eta(A_{0})$, $g_{A_{0}}(A_{1})\eta(A_{0})$ or $g_{A_{0}}(A_{2})\eta(A_{0})$ are always less than 1. One can see that the difference in in Eq. (S12) or (S13) is not related to the percent of lost habitat area.

Therefore, the change of fraction of unique species number after habitat loss is almost zero ($q_{before}-q_{after}\approx0$) in the logseries model when $k\to\infty$, i.e., the local community after losing habitat has a very minor effect on the fraction of unique species.

**References**

Zillio, T. & He, F. (2010). Modeling spatial aggregation of finite populations. *Ecology*, **91**, 3698–3706.

Kitzes, J. & Harte, J. (2015). Predicting extinction debt from community patterns. *Ecology*, **96**, 2127–2136.

**Additional figures**

Fig. S1. Prediction of extinction debt, immigration credit and fraction of unique species change for a single local community and a pair of local communities respectively in the remaining intact region. All scenarios here assume both SAD and SAAD are fixed before and after habitat loss. Species distribution is assumed to be extremely random (*k*=1000) or extremely aggregated (*k*=0.001). A-B: extinction debt or immigration credit as a function of area size of a single local community; C-D: extinction debt or immigration credit for endemic species in a single local community; E-F: extinction debt or immigration credit contributed by small-population species (the area of the local community is fixed to have a fraction 0.4 over the region); and G-H: fraction of unique species change as a function of area size of one local community in a pair (the area size of the other community is fixed to be 10% of the whole region). Before habitat destruction, the region is assumed to have 30 species and 200 individuals in the modeling. In all the subplots, dashed lines represent the outcomes when 50% of the region is destructed while solid lines represent the outcomes when only 10% of the region is destructed.

| A) | B) |
| --- | --- |
| C) | D) |
| E) | F) |
| G) | H) |

Fig. S2. Predicted extinction debt or immigration credit for non-endemic species in the single local community for lognormal and logseries models. In A and B, both SAD and SAAD are fixed; in C and D, SAAD is shifted while SAD is fixed. In all the subplots, aggregation distribution of species is assumed to have *k*=1 while random distribution of species is assumed to have *k* =100.

| A) | B) |
| --- | --- |
| C) | D) |
